# Clinical characteristics and immunological abnormalities of Castleman disease complicated with autoimmune diseases

**DOI:** 10.1007/s00432-020-03494-2

**Published:** 2021-02-05

**Authors:** Dao-Ping Sun, Wen-Ming Chen, Li Wang, Zhen Wang, Jin-Hua Liang, Hua-Yuan Zhu, Lei Fan, Yu-Jie Wu, Wei Xu, Jian-Yong Li

**Affiliations:** 1 Department of Hematology, The First Affiliated Hospital of Nanjing Medical University, Jiangsu Province Hospital, Nanjing, 210029 China; 2 Pukou CLL Center, Nanjing, 210000 China; 3 Department of Pathology, The First Affiliated Hospital of Nanjing Medical University, Jiangsu Province Hospital, Nanjing, 210029 China; 4 Department of Hematology, Jining No. 1 People’s Hospital, Jining, 272011 China; 5 Department of Oncology, Jining No. 1 People’s Hospital, Jining, 272011 China

**Keywords:** Castleman disease, Autoimmune diseases, Immunological abnormalities, T cells, Natural killer cells

## Abstract

**Purpose:**

To explore the clinical features and immunological mechanisms of Castleman disease (CD) complicated with autoimmune diseases (AID).

**Methods:**

We explored the prevalence and clinical manifestations of CD complicated with AID by reviewing clinical, pathological, and laboratory data of 40 CD patients retrospectively, and then explored abnormal immune mechanisms in the co-existence of the two entities by monitoring lymphocyte subsets in peripheral blood.

**Results:**

Paraneoplastic pemphigus, autoimmune hemolytic anemia, Sjogren’s syndrome, myasthenia gravis, and psoriasis were found to be coexisted with CD in 9/40 (22.5%) patients with different sequence of onset. No bias in the clinical and histological type of CD was observed for the occurrence of AID. CD patients with AID were more likely to have skin and/or mucous membrane damage and pulmonary complications, and presented elevated erythrocyte sedimentation rate, hypergammaglobulinemia, and positive autoantibodies than those without AID (*p* < 0.05). Deregulated cellular and innate immune responses as indicated by decreased CD3^+^ T cells and increased natural killer cells were observed in peripheral blood of CD patients with AID (*p* < 0.05). UCD patients with AID were successfully treated with surgery and immunosuppressive therapy. MCD complicated by AID relieved with immunosuppressors, cytotoxic chemotherapy, and rituximab.

**Conclusion:**

Systemic inflammation/immunological abnormalities and organ dysfunction were associated with the occurrence of AID in CD. Impairment of cellular and innate immunity may be a candidate etiology for the coexistence of the two entities.

## Introduction

Castleman disease (CD) represents a group of disorders characterized by polyclonal B lymphocyte proliferation, often associated with autoimmune manifestations, and occasionally be complicated with autoimmune connective tissue disease, such as systemic lupus erythematous (SLE), rheumatoid arthritis (RA), Sjogren’s syndrome (SS), mixed connective tissue disease, autoimmune hemolytic anemia (AIHA), myasthenia gravis (MG), amyloidosis, or thrombotic thrombocytopenic purpura (Muskardin et al. 2012; Wang et al. 2018; Ben-Chetrit et al. 1989; Lee et al. 2012; Yuzuriha et al. 2011; Tabata et al. 2019; Dei-Adomakoh et al. 2018; Ishikawa et al. 2014; Ogita et al. 2007; London et al. 2017). In addition, CD is related to the pathogenesis of paraneoplastic pemphigus (PNP) which is characterized by autoimmune-mediated cutaneous lesions with concomitant occurrence of either occult or confirmed systemic neoplasm (Bin et al. 2019). CD should be suspected when there is a change in features of a patient’s autoimmune disease (AID), or when the AID is unusually challenging to treat (Muskardin et al. 2012). However, due to limited numbers of case reports and studies on CD and associated AID, lack of profound understanding and extensive attention on the coexistence of these two entities may confound the clinical picture, resulting in delayed diagnosis and suboptimal treatment. Early identification of CD could be challenging, particularly in patients with AID that frequently manifest with lymphadenopathy (Muskardin et al. 2012). In-depth understanding of the prevalence and clinical manifestations of CD complicated with AID will be helpful to make an early and timely diagnosis.

The associations of CD with AID have not been fully illustrated. Autoimmune manifestations and autoantibodies are frequently found in CD patients without a definitive autoimmune diagnosis (Fajgenbaum et al. 2014; Liu et al. 2016). In reverse, several autoimmune disorders, such as SLE and RA, could present disseminated lymphadenopathy with histopathological findings compatible with CD (Kojima et al. 2000, 2006). These suggest that they likely share same features of pathophysiology. CD is an atypical lympho-proliferative disorder accompanied by severe chronic inflammatory responses (Fajgenbaum and Shilling 2018). B cells in germinal centers of hyperplastic lymph node continuously produce excessive interlukin-6 (IL-6), and this cytokine is responsible for the variety of clinical symptoms and laboratory abnormalities in patients with this disorder (Fajgenbaum and Shilling 2018; Yoshizaki et al. 2018). IL-6 may also be responsible for autoimmune phenomena associated with CD, by inducing expansion of autoantibody-producing CD5^+^ B lymphocytes (Muskardin et al. 2012). In reverse, autoantibodies may stimulate antigen-presenting cells in the lymph node to release more proinflammatory cytokines, resulting in exacerbation of CD symptoms (Fajgenbaum et al. 2014). However, it should be noted that, other cytokines and impairment of cellular immune mechanisms may also favor the development of both AID and CD (Marchi et al. 2004). Evaluation of their shared pathogenesis allows the development of more effective therapies.

In this study, we explored the prevalence and clinical manifestations of CD complicated with AID, and then explored abnormal immune mechanisms in the co-existence of the two entities by monitoring lymphocyte subsets in peripheral blood. Finally, we discussed the treatment and outcomes of CD accompanied by AID.

## Methods

### Patients

A total of 42 cases of histologically confirmed CD, including 22 males and 20 females, with a median age of 42 and a range of 15–80 years, were encountered at the First Affiliated Hospital of Nanjing Medical University between 2012 and 2019. The diagnosis of CD was established by excisional lymph node biopsy in all patients.

### Definitions

For the histopathology diagnosis of CD, microscopic slides from all patients were retrieved from the Pathology Department and reviewed by two pathologists. CD was divided into two major histological types, the hyaline vascular (HV) and plasma cell (PC) type, according to the criteria of Keller et al. (1972). For the clinical diagnosis, CD was divided into unicentric CD (UCD), which involved a single enlarged lymph node or region of lymph nodes and multicentric CD (MCD), which involved multiple lymph node stations (Dispenzieri and Fajgenbaum 2020). MCD is further divided into idiopathic MCD (iMCD), HHV8-associated MCD (HHV8-MCD), and POEMS-associated MCD (Dispenzieri and Fajgenbaum 2020).

Among patients with histologically confirmed CD, those who meet full criteria for autoimmune disorders including SS, psoriasis, MG, AIHA, and PNP were diagnosed as AID coexisting with CD (Stefanski et al. 2017; Sève et al. 2008; Kartan et al. 2017; Sehgal and Srivastava 2009; Raychaudhuri et al. 2014; Hehir and Silvestri 2018). As the majority of enlarged lymph nodes from patients with RA and 15–30% of SLE display MCD-like histopathology, patients with “Castleman-like” histopathological changes who fulfill the criteria for a definitive diagnosis of SLE and RA were excluded from the diagnosis of MCD (Fajgenbaum et al. 2017; Petri et al. 2012; Aletaha et al. 2010). Patients with autoantibodies who do not meet full criteria for a rheumatologic condition and have pathologic features and other criteria consistent with CD are diagnosed as CD.

### Data collection

A thorough review of systems, physical examination, complete blood count, urinalysis, erythrocyte sedimentation rate (ESR), C-reactive protein (CRP), rheumatoid factor (RF), anti-streptolysin O (ASO), Coombs test, liver function tests, serum creatinine, lactate dehydrogenase, serum immunoglobulin, serum protein electrophoresis with immunofixation, antinuclear antibody (ANA), double stranded antinuclear antibody (ds-DNA), extractable nuclear antigen polypeptide antibodies (ENA), antineutrophil cytoplasmic antibody (ANCA), anticardiolipin antibody (ACA), serology for HBV, HCV and HIV, and CT of chest, abdomen, and pelvis (or PET/CT) were done for all patients. Relevant clinical, pathological, and laboratory data were obtained by reviewing patients’ medical records and appropriately contacting identified patients by telephone conversations up to August 2019.

### Analysis of lymphocyte subsets

Analysis of peripheral blood lymphocyte subpopulations by flow cytometry was performed at the diagnosis of CD. Peripheral blood samples were collected and stained using the whole blood lysis technique. Phenotypic analyses were performed by flow cytometry using BD multitest CD3/CD8/CD45/CD4 reagent and CD3/CD16^+^ CD56/CD45/CD19 reagent. These antibodies are labeled as follows: PerCP-anti-CD45, FITC-anti-CD3, APC-anti-CD4, PE-anti-CD8, APC-anti-CD19, PE-anti-CD16 +CD56 (all from BD Biosciences USA). The percentages of the lymphocyte subsets including CD3^+^ T cells, CD4^+^ T cells, CD8^+^ T cells, CD19^+^ B cells, and CD3^−^CD16^+^CD56^+^ Natural killer (NK) cells, as well as CD4^+^/CD8^+^ ratio were measured.

### Statistical analysis

Continuous variables are expressed as mean ± standard deviation (SD) and categorical ones as number of cases (percentage). K–S test was used for normality test. Differences in numerical data were compared by the independent *t* test or Mann–Whitney *U* test. Differences in categorical data were compared using the chi-square test or Fisher exact test. Data analysis was performed using SPSS21 statistical software. A value of *p* < 0.05 was considered to be significant.

## Results

### Prevalence of CD complicated by AID

Except two patients with “Castleman-like” histopathological changes who fulfill the criteria for SLE and RA, respectively, were excluded from the diagnosis of CD, 40 patients were diagnosed as CD definitely. Among them, 25 patients were diagnosed with UCD, and 15 patients with MCD, including 13 iMCD, 1 HHV8-MCD, and 1 POEMS-associated MCD. Histological types included HV in 25 patients, and PC in 15 patients.

AID was diagnosed in 9/40 (22.5%) cases, including 4 cases of PNP, 2 cases of AIHA, and 1 case each of SS, MG, and psoriasis, with different sequence of onset—the diagnosis of SS and psoriasis were made preceded iMCD; AIHA in 2 iMCD patients and PNP in 3 UCD and 1 iMCD patients were diagnosed contemporaneously with CD; besides, MG occurred following the removal of tumor mass in one case with UCD (Table 1). The occurrence of AID in patients with MCD was higher than that in patients with UCD with no significant difference (33.3% vs. 16%, *p* = 0.255).

**Table 1:** The prevalence of AID in CD patients with different sequence of onset

Types of AID	UCD (*n* = *4*)	MCD (*n* = *5*)	AID occurred preceded CD (*n* = 2)	AID occurred concurrently with CD (*n* = 6)	AID occurred after CD (*n* = 1)
SS		1	1		
psoriasis		1	1		
AIHA		2		2	
PNP	3	1		4	
MG	1				1

*AID* autoimmune diseases, *CD* Castleman disease, *UCD* unicentric CD, *MCD* multicentric CD, *SS* Sjogren’s syndrome, *AIHA* autoimmune hemolytic anemia; *PNP* Paraneoplastic pemphigus, *MG* myasthenia gravis

### Clinical characteristics of CD patients with AID

Clinical features of CD patients complicated with AID are illustrated in Table 2. The median age of the cohort was 36 (16–59) years. Of the 9 patients, 48% were male, and 55.6% had MCD. The histology was PC variant in five cases and HV variant in four cases. Systemic manifestations were common in them. Multicentric lymphadenopathy was reported in 55.6% patients. The next frequently clinical features included B symptoms (33.3%), hepatomegaly and splenomegaly (22.2%), and edema, ascites, or pleural effusion (11.1%). No significant difference was observed in sex, age, clinical and pathological type of CD, and systemic manifestations for those patients as compared to those without AID (*p* > 0.05).

**Table 2:** Comparison of clinical characteristics between CD patients with AID (*n* = 9) and without AID (*n* = 31)

Characteristics	CD with AID (*n* = 9)	CD without AID (*n* = 31)	*p* value
Sex			1.000
Male, *n* (%)	5 (55.6)	17 (54.8)	
Female, *n* (%)	4 (44.4)	14 (45.2)	
Median age (range)	36 (16−59)	43 (15−80)	0.172
Clinical type			0.255
UCD, *n* (%)	4 (44.4)	21 (67.7)	
MCD, *n* (%)	5 (55.6)	10 (32.3)	
Pathological type			0.255
Hyaline vascular, *n* (%)	4 (44.4)	21 (67.7)	
Plasma cell, *n* (%)	5 (55.6)	10 (32.3)	
Systemic manifestations			
Night sweats, fever, and weight loss, *n* (%)	3 (33.3)	5 (16.1)	0.348
Multicentric lymphadenopathy, *n* (%)	5 (55.6)	10 (32.3)	0.255
Hepatomegaly and/or splenomegaly, *n* (%)	2 (22.2)	8 (25.8)	1.000
Edema, ascites, or pleural effusion, *n* (%)	1 (11.1)	4 (12.9)	1.000
Organ dysfunction			
Skin and mucous membrane damage, *n* (%)	7 (77.8)	2 (6.5)	< 0.001
Nervous and muscle injury, *n* (%)	1 (11.1)	1 (3.2)	0.404
Renal impairment, *n* (%)^a^	0 (0)	4 (12.9)	0.557
Pulmonary complications, *n* (%)^b^	5 (55.6)	2 (6.5)	0.003

*CD* Castleman disease, *AID* autoimmune diseases, *UCD* unicentric CD, *MCD* multicentric CD

^a^Elevated serum creatinine levels above the reference range or proteinuria above 3.5 g/24 h

^b^Including pulmonary infiltrates, restrictive lung disease, lymphoid interstitial pneumonitis, and obliterative bronchiolitis

Furthermore, by analyzing damage or dysfunction of specific organs or systems, it was found that the majority of patients with AID presented with skin and/or mucous membrane damage. Especially, extensive and refractory mucositis favoring the tongue and the labial and buccal mucosa was found in all four CD patients with PNP. In addition, nearly half of CD patients with AID presented pulmonary complications. Dyspnea caused by severe obstructive ventilation dysfunction and respiratory muscle paralysis occurred in CD patients with PNP, SS, and MG. Besides, general weakness due to positive antibody against acetylcholine receptor and severe nervous injury developed in the case of CD with MG. No renal impairment was observed. Compared with those without AID, CD patients with AID were more likely to have skin and/or mucous membrane damage (77.8% vs. 6.5%, *p* < 0.001), and pulmonary complications (55.6% vs. 6.5%, *p* = 0.003) (Table 2).

### Laboratory features of CD patients with AID

Laboratory data for CD patients complicated with AID is illustrated in Table 3. Anemia was found in 44.4% patients. Thrombocytosis was seen in 22.2% cases, whereas no thrombocytopenia was noted. Besides, 66.7% patients had elevated levels of ESR, and 44.4% cases had elevated levels of CRP, RF and ASO elevated in 22.2% patients, respectively. Hypergammaglobulinaemia was recorded in 66.7% individuals. Moreover, autoantibodies including ANA, ds-DNA, ENA, ANCA and ACA were positive in 44.4% patients, and Coombs test was positive in `33.3% cases.

**Table 3:** Comparison of laboratory abnormalities between CD patients with AID (*n* = 9) and without AID (*n* = 31)

Variable	CD with AID (*n* = 9)	CD without AID (*n* = 31)	*p* value
Anemia (< 125 g/l for males, < 115 g/l for females), *n* (%)	4 (44.4)	10 (32.3)	0.694
Thrombocytosis (> 300 × 10^9^/l), *n* (%)	2 (22.2)	6 (19.4)	1.000
Thrombocytopenia (< 125 × 10^9^/l), *n* (%)	0 (0)	3 (9.7)	1.000
Elevated ESR (> 20 mm/h), *n* (%)	6 (66.7)	7 (22.6)	0.038
Elevated CRP (> 10 mg/l), *n* (%)	4 (44.4)	7 (22.6)	.227
Elevated RF (> 30 IU/ml), *n* (%)	2 (22.2)	0 (0)	0.225
Elevated ASO (> 200 IU/ml), *n* (%)	2 (22.2)	4 (12.9)	0.602
Hypergammaglobulinemia, *n* (%)	6 (66.7)	8 (25.8)	0.044
Positivity of autoantibodies, *n* (%)	5 (55.6)	5 (16.1)	0.029
Positive ANA, ds-DNA, ENA, ANCA and/or ACA, *n* (%)	4 (44.4)	4 (12.9)	0.059
Positive coombs test, *n* (%)	3 (33.3)	2 (6.5)	0.065

*CD* Castleman disease, *AID* autoimmune diseases, *UCD* multicentric CD, *MCD* unicentric CD, *ESR* erythrocyte sedimentation rate, *CRP* C-reactive protein, *RF* Rheumatoid factor, *ASO* Anti-streptolysin O, *ANA* antinuclear antibody, *ds-DNA* double stranded antinuclear antibody, *ENA* extractable nuclear antigen polypeptide antibodies, *ANCA* antineutrophil cytoplasmic antibody, *ACA* anticardiolipin antibody

Compared with those without AID, CD patients with AID showed higher incidence of elevated ESR (66.7% vs. 22.6%, *p* = 0.038) and hypergammaglobulinemia (66.7% vs. 25.8%, *p* = 0.044). Additionally, a significant higher rate of positive ANA, ds-DNA, ENA, ANCA, ACA and/or positive coombs test was observed in them (55.6% vs. 16.1%, *p* = 0.029). (Table 3).

### Immune function monitoring

To further understand the immune pathogenesis of CD accompanied by AID, we analyzed the change of peripheral lymphocyte subsets in 6 CD patients with AID and 12 patients without AID at the diagnosis of CD.

As shown in Table 4, a significant lower proportion of CD3^+^ T cells (64.8 ± 11.4% vs. 74.6 ± 8.5%, *p* = 0.033) and a higher proportion of NK cells (25.7 ± 12.7% vs 11.7 ± 4.4%, *p* = 0.028) were observed in patients with AID. A tendency of fewer CD4^+^ T cells (29.6 ± 10.0% vs. 37.1 ± 7.0%, *p* = 0.062) was also observed in cases with AID. However, no significant difference in the percentage of CD8^+^ T cells and B cells subsets was found in two groups. The CD4^+^: CD8^+^ ratio was not significantly lower in patients with AID.

**Table 4:** Distribution of lymphocyte populations in peripheral blood of CD patients with AID (*n* = 6) and without AID (*n* = 12)

Lymphocyte subsets	CD with AID (*n* = 6)	CD without AID (*n* = 12)	*p* value
CD3^+^ T cells (%)	64.8 ± 11.4	74.6 ± 8.5	0.033
CD4^+^ T cells (%)	29.6 ± 10.0	37.1 ± 7.0	0.062
CD8^+^ T cells (%)	31.9 ± 9.5	35.8 ± 12.1	0.483
CD4^+^:CD8^+^ ratio	1.0 ± 0.6	1.2 ± 0.8	0.773
B cells (%)	7.9 ± 6.1	10.34 ± 2.8	0.289
Nature killer cells (%)	25.7 ± 12.7	11.7 ± 4.4	0.028

*CD* Castleman disease, *AID* autoimmune diseases

### Treatment and outcomes

Treatment of CD complicated with AID was mainly based on the clinical type of CD (Table 5).

**Table 5:** Treatment and outcomes of CD complicated with AID

No.	Sex, age	CD typing	AID	Treatment	Outcomes
1	F, 31	HV UCD	PNP	Tumor excision	Remission
2	M, 18	HV UCD	PNP	Tumor excision, GC, MTX, IVIG	Remission
3	F, 31	PC MCD	PNP	Tumor excision, GC, MTX	Remission
4	F, 16	HV UCD	PNP&OB	Tumor excision, GC, MTX, IVIG, Aza, thalidomide	Relapse
5	M, 37	HV UCD	MG	Tumor and thymus excision, pyridostigmine bromide, GC	Remission
6	F, 46	PC MCD	SS	GC, Aza, hydroxychloroquine, TGP	Remission
7	M, 46	PC MCD	AIHA	R-CHOP	Remission
8	M, 44	PC MCD	AIHA	R-CHOP, R-MINE, CPT	Remission
9	M, 59	PC MCD	Psoriasis	COP, thalidomide	Remission

*CD* Castleman disease, *AID* autoimmune diseases, *M* male, *F* female, *HV* hyaline vascular, *PC* plasma cell, *UCD* unicentric CD, *MCD* multicentric CD, *SS* Sjogren’s syndrome, *AIHA* autoimmune hemolytic anemia, *PNP* Paraneoplastic pemphigus, *OB* obliterative bronchiolitis*, MG* myasthenia gravis, *GC* glucocorticoid, *MTX* methotrexate, *IVIG* intravenous immune globulin, *Aza* azathioprine, *TGP* total glycosides of paeony, *R* rituximab, *CHOP* cyclophosphamide/vincristine/Adriamycin/prednisone, *MINE* mitoxantrone/ifosfamide/etoposide, *CPT* cyclophosphamide/prednisone/thalidomide, *COP* cyclophosphamide/vincristine/prednisone

Except one UCD patient with PNP (no. 1) was cured by tumor excision, other 3 patients with PNP and CD (no. 2, 3, 4) accepted effective immunosuppressive therapy with corticosteroid (GC), methotrexate (MTX), and intravenous immune globulin (IVIG) perioperatively. Unfortunately, one among them (no. 4) relapsed with severe oral mucositis and pulmonary destruction leading to obliterative bronchiolitis (OB) after treatment. Pulmonary injury with respiratory failure was relieved with IVIG again. Thalidomide and oral methylprednisolone were adopted for maintenance therapy. It should be alerted that, one UCD patient (no. 5) with mediastinal UCD, HV type, experienced a postoperative myasthenic crisis after tumor and thymus excision. His general condition improved soon with pyridostigmine bromide and methyl-prednisolone.

Symptoms of MCD patient with co-existing SS (no. 6) was improved with immunosuppressive agents (GC, azathioprine, hydroxychloroquine, and total glycosides of paeony). Rituximab in combination with cytotoxic chemotherapy was effective in one patient with MCD and associated AIHA (no. 7), but failed in another case with the same diagnosis (no. 8). This patient took oral cyclophosphamide-prednisone-thalidomide (CPT) regimen subsequently, improvement of anemia was observed. Besides, MCD occurred following Psoriasis was relieved with combination chemotherapy followed by maintenance therapy with thalidomide (no. 9).

## Discussion

To make an early and timely diagnosis of CD complicated with AID, we explored the prevalence of AID in a small population of patients with lymph node histopathological findings consistent with CD. AID was found to be coexisted with CD in 99/40 (22.5%) patients, a higher proportion than expected. Among them, PNP was most commonly observed. PNP is a mucocutaneous disease due to immunological effects of an obvious or occult lymphoproliferative disorder in most cases (Bin et al. 2019; Kartan et al. 2017; Sehgal and Srivastava 2009). CD constitute nearly 18.4% of hematologic neoplasms/disorder-related PNP (Sehgal and Srivastava 2009). We described four cases with CD associated PNP, and OB as a common and fatal compilation was noted in one of them. The second common AID associated with CD observed in our study was AIHA, which has been reported to be occasionally associated with MCD, although positive coombs test was more prevalent in this population (Liu et al. 2016). Besides, CD was also found to be complicated with SS in the present study. SS has also been described coexisting with CD in a few case reports, and CD may be a lymphoproliferative disorder associated with SS (Dei-Adomakoh et al. 2018). CD associated with MG is especially rare (Lee et al. 2012). This study described a postoperative myasthenic crisis in one patient with mediastinal UCD, HV type, which has been reported (Ishikawa et al. 2014). CD complicated by psoriasis observed in this study has not been reported. It should be noted that, AID may occur before, concurrent with, or after the diagnosis of CD, possibly with distinctive pathophysiology (Muskardin et al. 2012; Marchi et al. 2004).

We further explored the clinical characteristics of CD complicated with AID. No bias for the clinical and histological type of CD was observed for the occurrence of AID. Although MCD was found to be the form of CD, most frequently accompanied by symptoms and signs of autoimmunity in previous reports, and iMCD has a close association with several autoimmune and autoinflammatory diseases, UCD with localized lesions and less systematic syndromes could also be occasionally complicated with diverse autoimmune disorders (Muskardin et al. 2012; Fajgenbaum et al. 2014; Liu et al. 2016). Consistent with previous reports, AIHA and SS occurred in cases with PC variant, and MG in HV variant in this study (Yuzuriha et al. 2011; Tabata et al. 2019; Dei-Adomakoh et al. 2018; Ishikawa et al. 2014). In addition, the majority of PNP in our study was associated with HV type UCD as reported (Nikolskaia et al. 2003). An association between CD accompanied by AID and both systemic inflammation/immunological abnormalities and organ dysfunction was supported by our data. Compared with CD patients without AID, those with AID were more likely to have rash, mucous injury, and pulmonary complications. Moreover, elevated inflammatory markers ESR, hypergammaglobulinemia, and positive autoantibodies were more commonly observed in them. Hence, if rash, mucosal lesions, dyspnea, hypergammaglobulinemia or positive autoantibodies are present, further evaluation is required to exclude coexisting PNP, OB, and other autoimmune disorders in CD patients.

Mechanism for the coexistence of CD and autoimmune disorders in the same patient remained unclear. They likely share same features of pathophysiology. IMCD is speculated to manifest episodic systemic inflammatory symptoms, reactive proliferation of morphologically benign lymphocytes, and multiple-organ system impairment as a result of excessive interleukin-6 (IL-6) and other proinflammatory cytokines (Fajgenbaum et al. 2014). IL-6, as a multifunctional cytokine, may be responsible for autoimmune phenomena in iMCD by inducing the production of autoantibody and the expansion of autoantibody producing CD5^+^ B lymphocytes (Yoshizaki et al. 2018). Besides, IL-6 could also dysregulate the cellular immune response by inducing proliferation and differentiation of T cells (Kimura and Kishimoto 2010). This study investigated the cellular immune dysfunction by monitoring T cell subsets, and showed a significant decrease of CD3^+^ T cells and a tendency of fewer CD4^+^ T cells in peripheral blood of CD patients complicated with AID, indicating deregulated cellular immune responses, possible induced by IL-6, may be involved in its pathogenesis. Thus, impairment of cellular immune mechanisms, claimed to be an important determinant of the autoimmune complications in chronic lymphocytic leukemia (Hodgson et al. 2011) could operate in CD as well. In addition, increased NK cells observed in these patients suggested alterations in the innate immune system may also participant in the development of autoimmunity. Consistently, PNP which is associated with occult or confirmed hematologic and nonhematologic neoplasms may not be the result solely of a humoral response, autoreactive cellular autotoxicity mediated by CD8^+^ cytotoxic T lymphocytes, CD56^+^ NK cells, and CD68^+^ macrophages may also be implicated (Sehgal and Srivastava 2009).

It should be reminded that the biological mechanism for the frequency of autoimmunity in CD maybe complex. Evidence has been slowly accumulating for the role of additional cytokines, such as B-lymphocyte stimulator (BLyS), in the coexistence of AID and CD (Muskardin et al. 2012; Marchi et al. 2004). Better understanding of pathogenic cell types and cytokines will shed new light on its intricate pathogenesis. In addition, it is not clear if autoimmunity is the underlying cause or result of CD. Some autoimmune complications are known downstream complications of CD, such as PNP, AIHA, and MG, and some others are known upstream complications, such as SLE, SS, and RA, making CD a secondary disease (Muskardin et al. 2012; Marchi et al. 2004; Fajgenbaum et al. 2017). To clarify the distinctive pathophysiology between them, more efforts are needed to classify autoimmunity as downstream or upstream autoimmune diseases and analyze each group separately.

Understanding in CD and associated connective tissue diseases has allowed the development of targeted therapies. This study showed that UCD patients complicated with AID could be successfully treated with surgical excision of the tumor mass and immunosuppressive therapy. However, one case with PP and UCD relapsed with OB as a pulmonary compilation after treatment. Since perioperative immunosuppressive therapy is necessary to prevent fatal pulmonary injury in patients with CD and PNP (Nikolskaia et al. 2003), sustained and more powerful immunosuppressive treatment post-surgery might be needed for this patient. It should be alerted that, a rare postoperative myasthenic crisis may occur after the removal of tumor in patients with mediastinal solitary CD, early diagnosis guaranteed effective treatment in this study.

Currently, corticosteroids and/or combination chemotherapy were the most common interventions selected for patients with MCD (Liu et al. 2016). In this study, autoimmune manifestations relieved with immunosuppressors and cytotoxic chemotherapy in MCD patients with SS and Psoriasis, respectively. In addition, rituximab has also been effective for autoimmune cytopenia, with or without associated lymphoproliferative disorders, and may be particularly useful in patients with AID associated with MCD (Muskardin et al. 2012; Ocio et al. 2005). In this study, multidrug chemotherapy combined with rituximab was proved to be effective in one of the two MCD patients with AIHA. In another case with iMCD and AIHA, CPT regimen showed promising efficacy and safety as reported (Zhang et al. 2019). Besides, IL-6-based therapies have shown marked benefit in MCD and in a variety of AID. Treatment with Tocilizumab, a humanized anti-IL-6 receptor monoclonal antibody, resulted in either resolution or improvement of both CD and the AID (Yuzuriha et al. 2011; Dispenzieri and Fajgenbaum 2020). Hence, treatments that target pathogenic cell types, cytokines, or pathways that are shared in CD and associated AID would be more successful in the future.

Admittedly, cautious interpretation of our results is warranted because of several limitations of our retrospective study. Most importantly, we analyzed a small sample, sometimes with incomplete data. As a result, analyses were probably underpowered to identify the clinical and biological characteristics associated with AID in CD patients. Also, AID associated with CD are highly heterogeneous. Limited samples in this study constrained an in-depth analysis of the pathogenesis in patients with CD preceded or occurred contemporaneously with AID and those with AID preceded CD to clarify the distinctive pathophysiology between them.

In conclusion, this study analyzed the prevalence of AID in the cohort of CD patients, and identified systemic inflammation/immunological abnormalities and organ dysfunction associated with autoimmunity in CD. Impairment of cellular and innate immunity mechanisms may be a candidate etiology for the coexistence of the two entities. Early identification of CD and associated AID will guarantee more successful treatments.

## Data Availability

The datasets used and/or analyzed during the current study are available from the corresponding author on reasonable request. Code availability not applicable.
